# Association between perceived addiction and cessation behaviours among users of smokeless or combustible tobacco in India

**DOI:** 10.1111/dar.13507

**Published:** 2022-06-28

**Authors:** Vaibhav P. Thawal, Flora Tzelepis, Sima Ahmadi, Christine Paul

**Affiliations:** ^1^ School of Medicine and Public Health University of Newcastle Newcastle Australia; ^2^ Hunter Medical Research Institute Newcastle Australia; ^3^ Priority Research Centre for Health Behaviour University of Newcastle Newcastle Australia; ^4^ Hunter New England Population Health Hunter New England Local Health District Newcastle Australia; ^5^ Priority Research Centre for Cancer Research, Innovation and Translation University of Newcastle Newcastle Australia

**Keywords:** cessation, combustible tobacco, perceived addiction, smokeless tobacco

## Abstract

**Introduction:**

Addiction to tobacco is one of the main drivers of continued tobacco use. This study explored whether perceived addiction, type of tobacco and demographic characteristics were associated with past quit attempts (PQA), intention and self‐efficacy to quit among combustible tobacco (CT) or smokeless tobacco (SLT) users in India.

**Methods:**

A cross‐sectional survey was conducted among adult tobacco users (*N* = 607) attending an out‐patient department in Mumbai. Logistic regression analysis was used to investigate the association between demographic characteristics, type of tobacco, addiction perceptions and cessation behaviours.

**Results:**

Being ‘a little addicted’ (adjusted odds ratio, AOR [95% confidence interval, CI] 2.75 [1.83, 4.11], *P* < 0.0001) or ‘quite addicted’ (AOR [95% CI] 2.93 [1.53, 5.62], *P* < 0.0012) was associated with lower odds of making a PQA compared to ‘not addicted at all’. Being a SLT user (AOR [95% CI] 0.64 [0.41, 0.99], *P* = 0.047) and female (AOR [95% CI] 0.46 [0.26, 0.81], *P* = 0.0068) was associated with lower odds of making a PQA. There was a significant decrease in the odds of intention to quit as age increased by 1 year (AOR [95% CI] 0.98 [0.97, 0.99], *P* = 0.0018). Being identified as ‘a little addicted’ (AOR [95% CI] 0.28 [0.14, 0.55], *P* = 0.0003), ‘quite addicted’ (AOR [95% CI] 0.16 [0.07, 0.39], *P* < 0.0001) or ‘very addicted’ (AOR [95% CI] 0.09 [0.04, 0.19], *P* < 0.0001) was associated with lower odds of self‐efficacy to quit compared to ‘not addicted at all’.

**Discussion and Conclusion:**

Lack of awareness about addiction may inhibit cessation, particularly among less educated and female users of SLT and CT products.

**KEY POINTS**

- Perceived addiction to tobacco use is associated with past quit attempts.
- Perceived addiction to tobacco use is associated with self‐efficacy to quit.
- Intention to quit significantly decreases as age of tobacco users increases.
- Smokeless tobacco users have lower odds of past quit attempts.
- Female tobacco users have lower odds of past quit attempts compared to males.

## 1 INTRODUCTION

According to the Diagnostic and Statistical Manual of Mental Disorders, Fifth Edition, ‘tobacco addiction’ is the endorsement of at least 2 of 11 criteria (i.e. using in larger amounts or longer then intended; unsuccessful effort to cut down; time spent in obtaining or using; craving; recurrent tobacco use resulting in a failure to fulfil major role obligations at work, school or home; use despite having persistent or recurrent social or interpersonal problems caused by the effects of tobacco; important social, occupational or recreational activities given up or reduced; recurrent tobacco use in situations in which it is physically hazardous; use despite knowledge of the consequences; tolerance and withdrawal) in the past 12 months [1]. Use of combustible and smokeless tobacco products increase the risk of chronic diseases such as cancer and cardiovascular diseases and leads to addiction [2]. Addiction is an important reason for continued use despite the known negative consequences of use and intention to quit [3, 4].

A systematic review found that combustible tobacco (CT) users believe that CT use is addictive and that they are addicted to their smoking [5]. An important aspect of addiction as reported by CT users was lower control over smoking and difficulty in quitting [5]. The belief of being addicted to CT use may influence cessation behaviours, such that those who perceive being addicted to tobacco are motivated to stop smoking and make recent quit attempts compared to those who do not believe that they are addicted [3]. Some CT users acknowledge the role of addiction in their continued tobacco use while others deny being addicted [6]. CT users are sometimes ambivalent about being addicted [6] or express self‐exempting beliefs which minimise the harmful effects of CT use [7]. In a survey among CT users, a quarter believed that they could quit any time they wanted while one‐third believed that they were too addicted to quit [8]. Thus, understanding the association between addiction perceptions and cessation behaviour is highly relevant to tobacco control policy and to the practises of tobacco cessation services.

An exploration of the role of addiction perceptions in quitting behaviour must include a variety of behaviours which are associated with the eventual success or failure of a quit attempt [9]. These include past quit attempts (PQA), intention to quit and self‐efficacy to quit [9]. Tobacco users who have recently made a quit attempt are more likely to make another quit attempt and those who achieved longer abstinence during a quit attempt are more likely to successfully quit tobacco use in the next attempt [9]. Motivation or intention to quit tobacco are important predictors of successful cessation [10]. High self‐efficacy that is the confidence to quit is an important predictor for successful cessation [9].

Most of the available literature on association of perceived addiction on tobacco use and cessation behaviours has been conducted with CT users in developed countries; and there is a paucity of data on smokeless tobacco (SLT) users from less‐developed countries where most SLT use occurs [11]. SLT products contain tobacco, are not burned at the time of use and are used by chewing, keeping in the mouth or sniffing [12]. A range of SLT and CT products are considered socially acceptable and are commonly used in India including bidi smoking by males and chewing tobacco by females [13]. Please refer to Table S1, Supporting Information, for information on commonly used SLT products in India.

The Global Adult Tobacco Survey (GATS) 2 survey identified that a large proportion (92.7%) of current tobacco users in India believed tobacco use leads to addiction, however, it was unclear if there was any association of perceptions of addiction with PQAs, intention to quit and self‐efficacy to quit [14]. Data from the Tobacco Control policy survey found variation in addiction perceptions among users of different types of tobacco with a higher proportion (28%) of SLT users considering themselves not at all addicted to tobacco compared to CT users (16%) [15]. It is unclear if there are differences between CT and SLT users in how their perceptions about addiction may affect quitting behaviours. Some of the data on cessation behaviours in India showed that 38.5% of CT and 33.2% of SLT users made a quit attempt in the past 12 months [14]. This research also found that 55.4% of CT and 49.6% of SLT users reported an intention to quit in the future [14]. However, no studies in India have examined whether perceived addiction to tobacco products is associated with cessation behaviours.

Thus, it is important to investigate perceived addiction and cessation behaviour in non‐Western countries, in a context where both SLT and CT products are in common use. Understanding the association of perception of addiction and cessation behaviours may have important policy and practise implications. The aim of the study was to explore in an Indian context, whether type of tobacco use (CT or SLT), demographic characteristics (gender, age and education) and perceived level of addiction were associated with cessation behaviours (PQAs, intention to quit and self‐efficacy to quit).

## 2 METHODS

### 2.1 Design and settings

A cross‐sectional study was conducted in an out‐patient clinic of a 300‐bed multispecialty tertiary care government hospital in Navi Mumbai, Maharashtra, India between March and July 2019. The hospital catered to a population of 400,000 with an average of 1200 to 1300 patients a day visiting the outpatient department [16].

### 2.2 Participants

The study included patients attending the general medicine outpatient department who were 18 years or older, had used any form of tobacco (CT or SLT) in the past 30 days and who could speak Hindi, Marathi or English. Excluded participants were: (i) those using both SLT and CT [(a) to reduce the response burden of answering questions for multiple products, (b) to have clarity in response, and (c) dual users may use both CT and SLT products interchangeably, and it would be difficult for them to respond to a question on perception on addiction to a single tobacco product]; (ii) seeking treatment for tuberculosis, pregnant women in the third trimester of their pregnancy (were excluded to avoid any physical exertion that may be caused due to the study procedures); and (iii) those who were seriously ill (were unable to communicate with the research assistant due to illness). All identified current tobacco users were provided with self‐help materials with information on the health effects of using tobacco and the in‐house tobacco cessation service offered in the hospital. For those who participated in the survey, the information on the health effects of using tobacco and the in‐house cessation services were given after they completed the survey.

### 2.3 Procedure

Participants in the outpatient department waiting area were approached by a trained research assistant to share information about the study and assess eligibility. Eligible participants were provided with a participant information sheet in Hindi, Marathi or English as per their preference. Participants were further directed to a room in the outpatient department where the investigator was based to complete consent and the 15‐min survey in private. A pilot study with 43 participants was conducted to refine recruitment, data collection processes and the questionnaire. All participants interested in completing the survey provided written consent. For those with low levels of literacy the investigator read out the participant information sheet and consent form with consent indicated via a thumb impression on the consent form. The interviewer recorded responses using a computer tablet and all participants were given a Colgate toothpaste as a token of appreciation at the end of the survey. After completing the survey all participants were directed to a cessation counsellor for brief advice and participants interested in quitting tobacco were enrolled in a counselling program.

### 2.4 Measures

The survey was developed in English and further translated into Hindi and Marathi by an independent translation agency. After translation the survey was pilot tested with 43 participants which resulted in revisions being made to a few items before actual data collection. Data on sociodemographic characteristics and tobacco use patterns were collected using items form the GATS‐2 [14]. Items on addiction perceptions were adapted from the ‘Ontario Tobacco Research Unit—Questions from Population Surveys of Tobacco Use in Canada’ [17] and were modified to suit the survey population (Table 1). Items on cessation behaviours were adapted from the Tobacco Control Policy Survey India [15]. Measures and related items are presented in Table 1 in English, Hindi and Marathi.

**TABLE 1:** Measures and items (English, Hindi and Marathi)

Measures	Items
Socio‐demographic characteristics	Items on gender, age, highest level of education, marital status and main occupation over the past 12 months was collected.
Tobacco use	**The following item was used to identify current users of combustible and smokeless tobacco.** ‘Do you currently use any smoking or smokeless or both smoking and smokeless products?’ (Smoking products like cigarettes, bidi, hukkah and smokeless like khaini, betel quid, mawa, gutkha, mishiri etc.). Did you use any of the tobacco product in past 30 days? 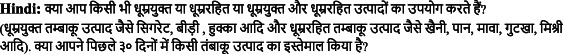 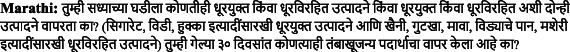 Additionally, items on type of tobacco product used (combustible, smokeless tobacco or both), form of tobacco product (smokeless tobacco products—betel quid with tobacco, khaini, gutka, mawa, snuff, mishri etc.; combustible products‐ cigarette, bidi, hukkah) and age of initiation were used.
Addiction perceptions	**The following item was used to explore addiction perceptions among tobacco users about perception of their own level of addiction:** (Instruction: When I use the word addictive or addiction, I mean being dependent on a substance like tobacco) 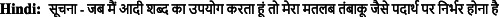 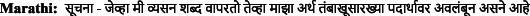 Original question from Population Surveys of Tobacco Use in Canada: How addicted to smoking cigarettes do you think you are? (a) Not addicted at all; (b) A little addicted; (c) Quite addicted; and (d) Very addicted. (The question was rephrased, and the word ‘smoking’ was replaced by tobacco to make it suitable for both combustible and smokeless tobacco users.) Adapted version: ‘Do you think you might be addicted to tobacco?’ with options (a) Not addicted at all; (b) A little addicted; (c) Quite addicted; and (d) Very addicted. *Note: In the translated Hindi and Marathi questionnaire, generally used terms for ‘addicted’ in India, that is* ‘ 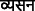 ’ *(vyasan) in Marathi and ‘* 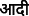 *’ (*aadee*) in Hindi*, *were used which are commonly used and understood by people in India*. 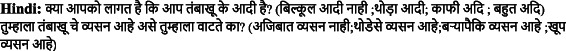
Past quit attempts	‘During the PAST 12 MONTHS, have you stopped using tobacco for one day or longer because you were trying to quit tobacco? (a) Yes; (b) No; (c) Do not know’ 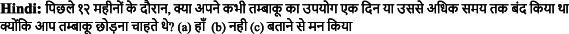 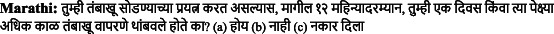
Intentions to quit	What best describes your intentions regarding quitting tobacco? Would you say you… (a) will quit in next 30 days; (b) will quit in next 6 months; (c) may quit in future, but not in next 6 months; (d) never expect to quit; and (e) do not know. [28] 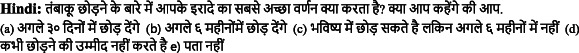 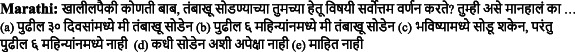
Self‐efficacy to quit	‘If you decide to give up tobacco use altogether, how likely do you think you would be to succeed? (a) very likely; (b) fairly likely; (c) fairly unlikely; (d) very unlikely; (e) do not know; and (f) refused’ [28]. 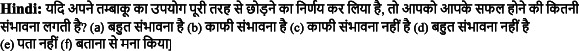 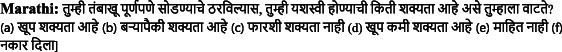

### 2.5 Statistical analysis

All data were checked for completeness and discrepancies before analysis. Statistical analyses were performed using SAS v9.4 (SAS‐Institute, USA). Data for one study participant whose survey was incomplete was excluded from the analysis. Descriptive statistics included frequencies and percentages for categorical variables, and means, SDs, medians and ranges for continuous variables. For the intention to quit item, the options were collapsed so that ‘Will quit in next 30 days’ and ‘Will quit in next 6 months’ were categorised as ‘Intention to quit’ and ‘May quit in the future, but not in next 6 months’ and ‘Never expect to quit’ was categorised as ‘no intention to quit in next 6 months’. For the item on self‐efficacy to quit, options ‘Very likely’ and ‘Fairly likely’ were categorised as ‘Self‐efficacy to quit’ and ‘Fairly unlikely’ and ‘Very unlikely’ were categorised as ‘low self‐efficacy to quit tobacco’. Crude and adjusted logistic regression analyses were used to assess the association between demographic characteristics (age, gender and education), perceived addiction and type of tobacco use (i.e. CT or ST) with cessation behaviours (i.e. PQAs, intention to quit and self‐efficacy). Statistical significance was set at *P*‐value <0.05.

### 2.6 Sample size

To achieve a significance level of 5% and 80% power, a sample size of 582 participants (291 CT and 291 SLT users) was required to detect a 10% difference in the perceptions about addiction among CT and SLT users.

## 3 RESULTS

**FIGURE 1:**
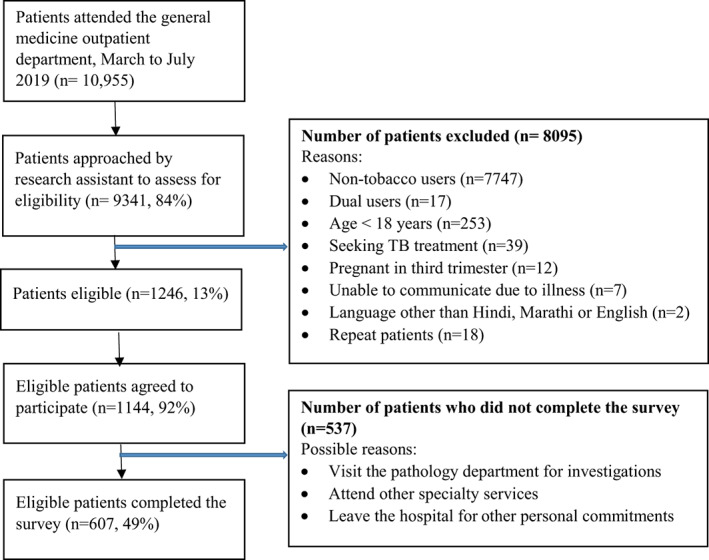
Recruitment flowchart

A total of 9341 (84%) of the 10,955 new patients attending the general medicine outpatient department were approached by the research staff to assess eligibility for the study. Please refer to the Figure 1 for flowchart on recruitment details. A total of 607 eligible participants completed the survey with an overall response rate of 49%. Some of the reasons for agreeing to participate but not completing the surveys were visit to the pathology department for investigations, had to attend other speciality services, had to leave for other personal commitments, etc. The demographic characteristics and tobacco use characteristics of participants are presented in Table 2. Table 3 presents the adjusted analysis of association for PQAs, intention and self‐efficacy to quit with demographic characteristics, perceived addiction and type of tobacco. Please refer to Table S2, for unadjusted regression model estimates in which perceived addiction, type of tobacco and demographic characteristics were modelled separately.

**TABLE 2:** Sociodemographic characteristics of participants

	Smokeless tobacco (*N* = 454)		Combustible tobacco (*N* = 153)		Total (*N* = 607)	
Characteristic	*n*	(%)	*n*	(%)	*n*	(%)
*Survey language*						
Marathi	231	50.9	63	41.2	294	48.4
Hindi	223	49.1	90	58.8	313	51.6
*Gender*						
Male	324	71.4	152	99.3	476	78.4
Female	130	28.6	1	0.7	131	21.6
*Age, years*						
18–24	38	8.4	21	13.7	59	9.7
25–44	244	53.7	70	45.8	314	51.7
45–64	156	34.4	53	34.6	209	34.4
65+	16	3.5	9	5.9	25	4.1
Mean (SD)	40.9	12.2	40.6	13.1	40.8	12.4
Median	39.5		40		40	
*Education level*						
No formal schooling	131	28.9	25	16.3	156	25.7
Primary school completed	108	23.8	60	39.2	168	27.7
Secondary and above	215	47.4	68	44.4	283	46.6
*Occupation*						
Government and non‐government employee	109	24	37	24.2	146	24.1
Self‐employed	239	52.6	98	64.1	337	55.5
Student	2	0.4	1	0.7	3	0.5
Home maker	80	17.6	17	11.1	97	16
Retired or unemployed	24	5.3	0	0	24	4
*Marital status*						
Single	41	9	27	17.7	68	11.2
Married	376	82.8	121	79.1	497	81.9
Divorced	1	0.2	0	0	1	0.2
Separated	9	2	0	0	9	1.5
Widowed	27	6	5	3.3	32	5.3
*Frequency of tobacco use*						
Daily	450	99.1	153	100	603	99.3
Less than daily	4	0.9	0	0	4	0.01
*Perceived addiction*						
Not all addicted	213	46.9	55	36	268	44.2
A little addicted	157	34.6	68	44.4	225	37.1
Quite addicted	37	8.1	13	8.5	50	8.2
Very addicted	47	10.4	17	11.1	64	10.5
*Intention to quit*						
Will quit in next 30 days	162	35.7	57	37.3	219	36.1
Will quit in next 6 months	61	13.4	24	15.7	85	14
May quit in future, but not in next 6 months	212	46.7	65	42.5	277	45.6
Never expect to quit	19	4.2	7	4.6	26	4.3
*Self‐efficacy to quit*						
Very likely	163	35.9	50	32.7	213	35.1
Fairly likely	205	45.2	81	52.9	286	47.1
Fairly unlikely	50	11	15	9.8	65	10.7
Very unlikely	11	2.4	3	2.0	14	2.3
Do not know	25	5.5	4	2.6	29	4.8

**TABLE 3:** Association of cessation behaviours (quit attempts, intention to quit and self‐efficacy to quit) with demographics, perceived addiction and type of tobacco (adjusted regression)

	Past quit attempts				Intention to quit				Self‐efficacy to quit			
Variable	No (*n* = 391)	Yes (*n* = 216)	Adjusted regression (*n* = 606) OR (95% CI)		No intention to quit in next 6 months (*n* = 303)	Intention to quit (*n* = 304)	Adjusted regression (*n* = 606) OR (95% CI)		Low self‐efficacy to quit (*n* = 79)	Self‐efficacy to quit (*n* = 499)	Adjusted regression (*n* = 577) OR (95% CI)	
*Perceived addiction*				<0.0001				0.8939				<0.0001
Not addicted at all	206 (77%)	62 (23%)	Reference		135 (50%)	133 (50%)	Reference		13 (5.0%)	245 (95%)	Reference	
A little addicted	117 (52%)	108 (48%)	2.75 (1.83, 4.11)	<0.0001	114 (51%)	111 (49%)	0.97 (0.67, 1.40)	0.8597	33 (15%)	181 (85%)	0.28 (0.14, 0.55)	0.0003
Quite addicted	26 (52%)	24 (48%)	2.93 (1.53, 5.62)	0.0012	25 (50%)	25 (50%)	0.99 (0.53, 1.83)	0.9756	11 (23%)	36 (77%)	0.16 (0.07, 0.39)	<0.0001
Very addicted	42 (66%)	22 (34%)	1.49 (0.81, 2.73)	0.1956	29 (45%)	35 (55%)	1.21 (0.69, 2.12)	0.5041	22 (37%)	37 (63%)	0.09 (0.04, 0.19)	<0.0001
*Type of tobacco*												
Smokeless only	307 (68%)	147 (32%)	0.64 (0.41, 0.99)	0.0470	231 (51%)	223 (49%)	0.77 (0.51, 1.18)	0.2343	61 (14%)	368 (86%)	0.65 (0.33, 1.26)	0.2013
Smoking only	84 (55%)	69 (45%)	Reference		72 (47%)	81 (53%)	Reference		18 (12%)	131 (88%)	Reference	
*Gender*												
Female	104 (79%)	27 (21%)	0.46 (0.26, 0.81)	0.0068	68 (52%)	63 (48%)	0.91 (0.56, 1.46)	0.6847	16 (14%)	100 (86%)	0.61 (0.28, 1.32)	0.2082
Male	287 (60%)	189 (40%)	Reference		235 (49%)	241 (51%)	Reference		63 (14%)	399 (86%)	Reference	
*Age*												
Mean (SD)	41.06 (12.42)	40.33 (12.40)	1.00 (0.99, 1.02)	0.8953	42.61 (12.04)	39.00 (12.54)	0.98 (0.97, 0.99)	0.0018	40.58 (12.76)	40.48 (12.32)	1.00 (0.98, 1.02)	0.9605
Median (min, max)	40.00 (18.00, 77.00)	39.00 (18.00, 69.00)			42.00 (18.00, 77.00)	37.00 (18.00, 73.00)			38.00 (21.00, 73.00)	40.00 (18.00, 77.00)		
*Education level*				0.0016				0.5392				0.1363
No formal schooling	122 (78%)	34 (22%)	Reference		80 (51%)	76 (49%)	Reference		12 (8.3%)	132 (92%)	Reference	
Primary school completed	115 (69%)	52 (31%)	1.49 (0.87, 2.53)	0.1451	90 (54%)	77 (46%)	0.81 (0.51, 1.27)	0.3504	23 (14%)	138 (86%)	0.49 (0.22, 1.06)	0.0713
Secondary and above	154 (54%)	129 (46%)	2.36 (1.45, 3.83)	0.0005	132 (47%)	151 (53%)	0.99 (0.65, 1.51)	0.9597	44 (16%)	228 (84%)	0.50 (0.24, 1.04)	0.0644

CI, confidence interval; OR, odds ratio.

### 3.1 Participant characteristics and tobacco use factors

As reported in Table 2, the mean age of participants was 40.8 (SD = 12.41) years and age range 18 to 77 years. Mean age of tobacco use initiation was 18.95 (SD = 6.54) years. Majority (78%) of the sample was male. Nearly half (53%) of the participants had a primary school or lower education. Current use of CT products was reported by 25% while 75% reported using SLT products.

### 3.2 Characteristics associated with quit attempts

Participants who reported being ‘A little addicted’ had significantly higher odds of making a PQAs (adjusted odds ratio; AOR [95% confidence interval; CI] 2.75 (1.83, 4.11), *P* < 0.0001) as did participants who reported being ‘Quite addicted’ (AOR [95% CI] 2.93 [1.53, 5.62], *P* = 0.0012) compared to those who perceived they were ‘Not addicted at all’ (Table 3). SLT users compared to CT users (AOR [95% CI] 0.64 [0.41, 0.99], *P* = 0.0470) and female participants compared to males (AOR [95% CI] 0.46 [0.26, 0.81], *P* = 0.0068) had significantly lower odds of making a PQA. Participants with a ‘Secondary and above’ education level had significantly higher odds of a PQA compared to those with ‘No formal education’ (AOR [95% CI] 2.36 [1.45, 3.83], *P* = 0.0005).

### 3.3 Characteristics associated with intention to quit

Table 3 presents the factors associated with intention to quit. There was a significant decrease in the odds of intention to quit as age increased by 1 year (AOR [95% CI] 0.98 [0.97, 0.99], *P* = 0.0018) (Table 3). Similarly, a higher proportion of males (51%) compared to females (48%) reported having an intention to quit, however, the differences were not statistically significant.

### 3.4 Characteristics associated with self‐efficacy to quit

As reported in Table 3 in the adjusted analysis, compared to those who reported they were ‘Not addicted at all’ the odds of self‐efficacy to quit was significantly lower among participants who identified themselves as ‘A little addicted’ (AOR [95% CI] 0.28 [0.14, 0.55], *P* = 0.0003), ‘Quite addicted’ (AOR [95% CI] 0.16 [0.07, 0.39], *P* < 0.0001) or ‘Very addicted’ (AOR [95% CI] 0.09 [0.04, 0.19], *P* < 0.0001).

## 4 DISCUSSION

This study found that perceived addiction to tobacco was associated with quit attempts in the past 12 months and self‐efficacy to quit but not with intention to quit in the future. These findings are an important addition to the literature on addiction perceptions and cessation behaviours, the majority of which has come from developed countries where tobacco use is predominately in the form of cigarettes [3]. Thus, the study fills the large gap in knowledge regarding addiction perceptions and how they are associated with cessation behaviours by tobacco users in India.

Some of the notable findings of our study include identification of only one female using CT and use of only SLT products by retired or unemployed people. Use of CT products by females is unacceptable in India [18] and is one of the reason for low prevalence (1.7%) of CT use among females in India [14]. Further, the cost of tobacco is important to product preferences and SLT is generally cheaper compared to cigarettes, which could explain why retired or unemployed people reported using only SLT products [19].

Consistent with a previous study with CT users [3], our findings indicated that perceived addiction was associated with reported PQAs. These findings suggest that tobacco users who do not perceive themselves as a little or quite addicted to tobacco are less likely to have made PQAs. Although tobacco (SLT and CT) users view themselves as addicted to tobacco, our study confirms that there are still many (47% SLT users and 36% CT users) who do not believe they are addicted to tobacco. Such tobacco users may possess risk‐minimising beliefs about being addicted to tobacco use and may be less likely to make quit attempts [7]. Oakes *et al*. classified such beliefs as ‘bulletproof’ beliefs in which tobacco users possess self‐exemptions that they have good health or genes and that the health effects of tobacco do not apply to them [7, 20]. Our results on PQAs were similar to the findings from the GATS‐1 survey such that higher educational attainment and gender were associated with PQAs [13]. Specifically, in the GATS‐1 survey, compared with those with no formal education, those with secondary education level had significantly higher odds of making quit attempts and males had significantly higher odds than females of making quit attempts [13]. Similar to available literature [15], our study found that compared to CT users, SLT users had significantly lower odds of making a PQA. Some of the known reasons that help explain this finding include SLT being culturally acceptable, normative attitudes towards SLT and lack of understanding on the health effects and addictiveness of SLT products [21]. These findings suggest that targeted awareness campaigns about the addictive potential of tobacco products are required, and that it is important that such campaigns specifically mention SLT. Pictorial health warnings that depict the addictive potential of different tobacco products may be particularly important to inform tobacco users with low literacy levels. Although a past study assessing the impact of pictorial warnings showed that these warnings are effective in educating tobacco users and helping in reducing or quitting tobacco use [22] the existing pictorial warnings on packages of CT and SLT do not carry information on addictive potential of tobacco products [23]. In addition to the population level strategies, individual level strategies, for example, culturally appropriate tobacco cessation interventions that make use of evidence‐based counselling techniques to clarify the misconceptions about addictive potential of SLT products would be required to change the perceptions of tobacco users in India.

Our study found that there was no statistically significant association of perceived addiction with intention to quit. Although, a higher proportion of CT users (53%) compared to SLT users (49%) reported having an intention to quit, the differences were not statistically significant. Our analysis showed that there was a statistically significant decrease in intention to quit as age increased. A similar trend of decline in level of willingness to quit with increasing age was observed in studies [24, 25] using data from the GATS‐1 and GATS‐2 surveys. These studies [24, 25] showed that participants in younger age groups had higher willingness to quit compared to older adults. These findings highlight the need for tobacco control and cessation strategies which leverage older age groups as tobacco users in younger age groups' have shown to have higher willingness to quit [25]. Our findings also suggest the need for further research to understand lower intention to quit among older tobacco users in India as a basis for developing strategies that could be used to increase intention to quit among this age group.

Our findings show that there was a significant association of perceived addiction with self‐efficacy to quit. Participants who perceived themselves to be a little addicted, quite addicted or very addicted to tobacco had lower self‐efficacy regarding successfully quitting compared to those who perceived themselves to be not addicted at all. Research shows that self‐efficacy has a significant relationship with quitting tobacco and preventing relapse [26]. Self‐efficacy plays a very important role in cognitive behaviour change among tobacco users attempting to quit [26] and thus it is necessary to provide evidence‐based counselling interventions which supports self‐efficacy in the face of addiction (e.g. the use of pharmacotherapies and environmental restructuring).

The study had a number of limitations. The use of the term ‘addiction’ when used in the context of tobacco use may perhaps be associated with shame, stigma or a bias [27]. This may have influenced responses and possibly resulted in a lower proportion of participants considering themselves addicted to SLT and CT products. The survey was conducted in a single tertiary care hospital catering to a socioeconomically diverse population from urban regions and thus the study findings may not be generalisable to rural regions of India. On comparing the characteristics of our sample to tobacco users from GATS‐2 survey we observed that females, participants aged 65 years and above, those with no formal education, those who reported completing primary school, current students and currently unemployed people were underrepresented in our sample [14, 25]. Therefore, future research should use strategies that may increase sample representativeness to address this limitation. Please refer to Table S3, for comparison of sample characteristic. Current tobacco use, PQAs, intention to quit and self‐efficacy to quit were self‐reported and therefore underreporting or misreporting due to social desirability bias may have occurred. A moderate participation rate in the survey is another limitation of the study. Another limitation is the imbalance in the number of SLT and CT users in our study. Although, the sample size calculation was based on an equal number of SLT and CT users, the number of CT users recruited was less than expected while more than expected SLT users were recruited.

This study has important policy and practise implications to prevent future tobacco attributable diseases and to reduce inequalities among vulnerable groups. First, it is important to create awareness in India about the addictive potential of both CT and SLT products through awareness campaigns to help inform tobacco users of the addictive potential of tobacco products and motivate them to reduce or quit tobacco completely. These campaigns should be designed with older individuals, female tobacco users and those with low literacy levels in mind. Awareness about the addictive potential of all forms of tobacco products should be created using mass media campaigns and through warning labels on tobacco packages. Culturally appropriate tobacco cessation interventions including awareness components that would address the inaccurate perceptions of tobacco addiction need to be designed. For tobacco users who acknowledge that they are addicted to their tobacco use and have low self‐efficacy to quit, evidence‐based counselling interventions should be provided in an effort to motivate them to quit successfully. Such interventions need to be delivered at community level in a way that may promote accessibility and acceptability and maximise participation from the vulnerable groups. Wider awareness in India of the addiction potential of tobacco products may help in prevent the uptake of tobacco use and motivate existing users to quit.

## 5 CONCLUSION

This study found that perceived addiction to tobacco was associated with PQAs and self‐efficacy to quit in India. These findings suggest that poor understanding of tobacco addiction has an inhibiting effect on cessation behaviours, particularly for SLT. Further research studying the association between addiction perceptions and cessation behaviours with a larger representative sample with wide range of sociodemographic characteristics is needed to build upon and expand on our study findings. If tobacco users do not perceive themselves to be addicted to tobacco, it may influence whether they make quit attempts and their self‐efficacy to quit. It is important that targeted efforts are made to fully and appropriately inform tobacco users about the addictive potential of all type of tobacco products.

## AUTHOR CONTRIBUTIONS

Vaibhav Thawal: Conceptualisation; data curation; investigation; methodology; project administration; roles/writing – original draft; writing – review and editing. Flora Tzelepis: Conceptualisation; data curation; investigation; methodology; project administration; supervision; roles/writing – original draft; writing – review and editing. Sima Ahmadi: Formal analysis; writing – review and editing. Christine Paul: Conceptualisation; data curation; investigation; methodology; project administration; supervision; roles/writing – original draft; writing – review and editing. All authors have read and approved the final version of the manuscript.

## FUNDING INFORMATION

Vaibhav Thawal was supported by a UNIPRS and UNRS Central 50:50 University of Newcastle PhD Scholarship. Flora Tzelepis was supported by a National Health and Medical Research Council Career Development Fellowship (APP1143269). Financial support for data collection and analysis was received from the Priority Research Centre for Health Behaviour, College of Health, Medicine and Wellbeing, University of Newcastle, small grants programme.

## CONFLICT OF INTEREST

The authors declare that they have no known competing financial interests or personal relationships that could have appeared to influence the work reported in this paper.

## ETHICS STATEMENT

Ethical approval was obtained from the Human Research Ethics Committee, University of Newcastle, Newcastle, Australia (Reference number‐HREC‐H‐2018‐0401) and Joint Ethics Committee, Narotam Sekhsaria Foundation‐Salaam Bombay Foundation, Mumbai, India (Reference number‐JEC/NSF‐SBF/2019/02).

## Supporting information


**Appendix S1** Supporting Information

**Table S1** Common smokeless tobacco products used in India
**Table S2** Association of cessation behaviours (quit attempts, intention to quit and self‐efficacy to quit) with demographics, perceived addiction and type of tobacco (unadjusted regression)
**Table S3** Comparison of sample characteristics with characteristics of tobacco users from GATS‐2 survey
