# Supplementary material for: Association between perceived addiction and cessation behaviours among users of smokeless or combustible tobacco in India
Source: Drug Alcohol Rev. 2022 Jun 28;41(7):1510–20. doi: 10.1111/dar.13507 (PMC9796830; doi:10.1111/dar.13507)
Supplement: Supplementary file 2 — Table S1 Common smokeless tobacco products used in India Table S2 Association of cessation behaviours (quit attempts, intention to quit and self‐efficacy to quit) with demographics, perceived addiction and type of tobacco (unadjusted regression) Table S3 Comparison of sample characteristics with characteristics of tobacco users from GATS‐2 survey [file DAR-41-1510-s002.docx]

**Table S1. Common smokeless tobacco products used in India^[[1]](#footnote-1)^**

| **Tobacco product** | **Details** |
| --- | --- |
| **Paan/betel quid with tobacco** | Ingredients – Betel leaf, areca nut, slaked lime, catechu and other flavouring agents such as menthol, camphor, sugar, rosewater, aniseed, cardamom, clove, mint, spices.  Mode of use – Placed in the mouth and chewed. |
| **Khaini** | Ingredients – Sun-dried or fermented coarsely cut tobacco leaves mixed with slaked lime.  Mode of use – Placed in the mouth between the gums and cheeks and sucked slowly for 10-15 minutes. Occasionally, left overnight in the mouth. |
| **Guthka** | Ingredients – Areca nut, slaked lime, catechu and sun-dried, roasted, finely chopped tobacco with flavourings and sweeteners.  Mode of use – Held in the mouth, sucked and chewed. |
| **Mishri** | Ingredients –It is roasted and powdered tobacco.  Mode of use –Applied to the gums using a finger, used as a dentifrice. |
| **Mawa** | Ingredients –It is a mixture of thin shavings of areca nut with some tobacco flakes and slaked lime.  Mode of use –Placed in the mouth and chewed for 10-20 minutes. |
| **Zarda** | Ingredients –Flavoured chewing tobacco flakes mixed with aromatic spices, menthol, herbs, fragrances, saffron, raw kiwam, silver flakes and sandalwood oil. Lime is provided separately by the manufactures.  Mode of use –Used in betel quid, chewed alone or mixed with lime or areca nut. |
| **Kharra** | Ingredients –Combination of tobacco, areca nut, lime, catechu with additional ingredients.  Mode of use –Held in the mouth, sucked and chewed. |

**Table S2. Association of cessation behaviours (quit attempts, intention to quit and self-efficacy to quit) with demographics, perceived addiction and type of tobacco (unadjusted regression)**

|  | **Past quit attempts** | | | | **Intention to quit** | | | | **Self-efficacy to quit** | | | | |
| --- | --- | --- | --- | --- | --- | --- | --- | --- | --- | --- | --- | --- | --- |
| **Variable** | **No** (n=391) | **Yes** (n=216) | **Unadjusted regression**  (n=606)  OR (95% CI) | | **No intention to quit in next 6 months**  (n=303) | **Intention to quit** (n=304) | **Unadjusted regression**  (n=606)  OR (95% CI) | | **Low self-efficacy to quit** (n=79) | **Self-efficacy to quit** (n=499) | **Unadjusted regression**  (n=577)  OR (95% CI) | |  |
| *Perceived addiction* |  |  |  | **<0.0001** |  |  |  | 0.8932 |  |  |  | <.0001 |  |
| Not addicted at all | 206 (77%) | 62 (23%) | Reference |  | 135 (50%) | 133 (50%) | Reference |  | 13 (5.0%) | 245 (95%) | Reference |  |  |
| A little addicted | 117 (52%) | 108 (48%) | 3.07  (2.08, 4.51) | **<0.0001** | 114 (51%) | 111 (49%) | 0.99  (0.69, 1.41) | 0.9482 | 33 (15%) | 181 (85%) | 0.29  (0.15, 0.57) | **0.0003** |  |
| Quite addicted | 26 (52%) | 24 (48%) | 3.07  (1.64, 5.72) | **0.0004** | 25 (50%) | 25 (50%) | 1.02  (0.55, 1.86) | 0.9614 | 11 (23%) | 36 (77%) | 0.17  (0.07, 0.42) | **<0.0001** |  |
| Very addicted | 42 (66%) | 22 (34%) | 1.74  (0.97, 3.14) | 0.0651 | 29 (45%) | 35 (55%) | 1.23  (0.71, 2.12) | 0.4673 | 22 (37%) | 37 (63%) | 0.09  (0.04, 0.19) | **<0.0001** |  |
| *Type of tobacco* |  |  |  |  |  |  |  |  |  |  |  |  |  |
| Smokeless only | 307 (68%) | 147 (32%) | 0.58  (0.40, 0.85) | **0.0047** | 231 (51%) | 223 (49%) | 0.86  (0.59, 1.24) | 0.4141 | 61 (14%) | 368 (86%) | 0.83  (0.47, 1.45) | 0.5132 |  |
| Smoking only | 84 (55%) | 69 (45%) | Reference |  | 72 (47%) | 81 (53%) | Reference |  | 18 (12%) | 131 (88%) | Reference |  |  |
| *Gender* |  |  |  |  |  |  |  |  |  |  |  |  |  |
| Female | 104 (79%) | 27 (21%) | 0.39  (0.25, 0.63) | **<0.0001** | 68 (52%) | 63 (48%) | 0.90  (0.61, 1.33) | 0.6070 | 16 (14%) | 100 (86%) | 0.99  (0.55, 1.78) | 0.9649 |  |
| Male | 287 (60%) | 189 (40%) | Reference |  | 235 (49%) | 241 (51%) | Reference |  | 63 (14%) | 399 (86%) | Reference |  |  |
| *Age* |  |  |  |  |  |  |  |  |  |  |  |  |  |
| Mean (SD) | 41.06 (12.42) | 40.33 (12.40) | 1.00  (0.98, 1.01) | 0.4845 | 42.61 (12.04) | 39.00 (12.54) | 0.98  (0.96, 0.99) | **0.0004** | 40.58 (12.76) | 40.48 (12.32) | 1.00  (0.98, 1.02) | 0.9460 |  |
| Median (min, max) | 40.00  (18.00, 77.00) | 39.00  (18.00, 69.00) |  |  | 42.00  (18.00, 77.00) | 37.00  (18.00, 73.00) |  |  | 38.00  (21.00, 73.00) | 40.00 (18.00, 77.00) |  |  |  |
| *Education level* |  |  |  | **<0.0001** |  |  |  | 0.3043 |  |  |  | 0.0902 |  |
| No formal schooling | 122 (78%) | 34 (22%) | Reference |  | 80 (51%) | 76 (49%) | Reference |  | 12 (8.3%) | 132 (92%) | Reference |  |  |
| Primary school completed | 115 (69%) | 52 (31%) | 1.62  (0.98, 2.68) | 0.0587 | 90 (54%) | 77 (46%) | 0.90  (0.58, 1.39) | 0.6388 | 23 (14%) | 138 (86%) | 0.55  (0.26, 1.14) | 0.1073 |  |
| Secondary and above | 154 (54%) | 129 (46%) | 3.01  (1.92, 4.70) | **<0.0001** | 132 (47%) | 151 (53%) | 1.20  (0.81, 1.78) | 0.3521 | 44 (16%) | 228 (84%) | 0.47  (0.24, 0.92) | **0.0284** |  |

CI, confidence interval, OR; odds ratio.

**Table S3. Comparison of sample characteristics with characteristics of tobacco users from GATS-2 survey^[[2]](#footnote-2)^**

Sociodemographic factors of tobacco users aged 15 years and above based on Global Adult Tobacco Survey India data, 2016-17 (n=21,085) compared to study sample (n=607)

|  | GATS-2 | | Study sample | |
| --- | --- | --- | --- | --- |
|  | n | % | n | % |
| *Age, years* |  |  |  |  |
| 18-24* | 1772 | 8% | 59 | 9.7% |
| 25-44 | 10432 | 49% | 314 | 51.7% |
| 45-64 | 6723 | 32% | 209 | 34.4% |
| 65+ | 2158 | 10% | 25 | **4.1%** |
| Total | 21085 |  |  |  |
| *Gender* |  |  |  |  |
| Female | 6026 | 29% | 131 | **21.6%** |
| Male | 15059 | 71% | 476 | 78.4% |
| Total | 21085 |  |  |  |
| *Education* |  |  |  |  |
| No formal education | 6654 | 32% | 156 | **25.7%** |
| Primary school completed | 9833 | 47% | 168 | **27.7%** |
| Secondary and above | 4589 | 22% | 283 | 46.6% |
| Total | 21076 |  |  |  |
| *Occupation* |  |  |  |  |
| Self employed | 6770 | 33% | 337 | 55.5% |
| Government/non-government employee | 2611 | 13% | 146 | 24.1% |
| Student | 6164 | 30% | 3 | **0.5%** |
| Unemployed | 4534 | 22% | 24 | **4%** |
| Homemaker | 403 | 2% | 97 | 16% |
| Total | 20482 |  |  |  |

*Age range for this subgroup was 15-24 years for GATS-2 survey. GATS, Global Adult Tobacco Survey.

1. Indian Council for Medical Research. National Institute of Cancer Prevention and Research. WHO Framework Convention on Tobacco Control. Commonly used Smokeless Tobacco Products around the globe. Available from: <https://untobaccocontrol.org/kh/smokeless-tobacco/paan-betel-quid-tobacco/> (Accessed 15 November 2021) [↑](#footnote-ref-1)
2. Kar S, Sivanantham P, Rehman T, Chinnakali P, Thiagarajan S. Willingness to quit tobacco and its correlates among Indian tobacco users—Findings from the Global Adult Tobacco Survey India, 2016–17. J Postgrad Med 2020;66:141-8. [↑](#footnote-ref-2)
